# Direct and Indirect Associations of Media Use With COVID-19 Vaccine Hesitancy in South Korea: Cross-sectional Web-Based Survey

**DOI:** 10.2196/32329

**Published:** 2022-01-06

**Authors:** Minjung Lee, Myoungsoon You

**Affiliations:** 1 Institute of Health and Environment Seoul National University Seoul Republic of Korea; 2 Office of Dental Education School of Dentistry Seoul National University Seoul Republic of Korea; 3 Department of Public Health Sciences Graduate School of Public Health Seoul National University Seoul Republic of Korea

**Keywords:** COVID-19, coronavirus, vaccination, vaccine hesitancy, media use, social media, public health, pandemic, epidemiology, online information, health information

## Abstract

**Background:**

The battle against the 2019 novel coronavirus (COVID-19) has not concluded. Despite the availability of vaccines, the high prevalence of vaccine hesitancy represents a significant challenge to public health, and raising vaccine acceptance among the public is critical. Although media has become an increasingly popular source of COVID-19 vaccine-related information, the question of whether and how media use is related to the public’s vaccine hesitancy warrants exploration.

**Objective:**

This study aimed to (1) examine the level of COVID-19 vaccine hesitancy, (2) identify factors associated with COVID-19 vaccine hesitancy, and (3) explore the direct and indirect relationship between media use and vaccine hesitancy through psychological factors.

**Methods:**

A month before COVID-19 vaccination was initiated in South Korea, we conducted a cross-sectional web-based survey over 6 days (January 20-25, 2021). This study included 1016 participants, and a logit model for regression analyzed associations between sociodemographic factors, health-related factors, psychological factors, and media use toward one’s COVID-19 vaccine hesitancy. Additionally, we conducted a path analysis to examine the indirect effects of media use on vaccine hesitancy by using psychological factors (ie, perceived risk of COVID-19 infection, perceived benefits, and perceived barriers of COVID-19 vaccination).

**Results:**

Among the participants (N=1016), 53.3% (541/1016) hesitated to take the COVID-19 vaccine, while 46.7% (475/1016) agreed to accept the vaccine. Of the sociodemographic factors, female gender (odds ratio [OR] 1.967, 95% CI 1.36-2.86; *P*<.001), age in 50s (OR 0.47, 95% CI 0.23-0.96; *P*=.004), and age over 60s (OR 0.49, 95% CI 0.24-0.99; *P*=.04) were significant individual predictors of COVID-19 vaccine hesitancy. Perceived susceptibility of infection (OR 0.69, 95% CI 0.52-0.91; *P*=.01) and perceived benefits of vaccination (OR 0.69, 95% CI 0.52-0.91; *P*=.01) were associated with lower vaccine hesitancy. Perceived barriers of vaccination (OR 1.63, 95% CI 1.29-2.07; *P*<.001) and lower trust in government (OR 0.72, 95% CI 0.53-0.98; *P*=.04) were related to vaccine hesitancy. The use of offline and online media as sources for the perceived benefits of vaccination was associated with vaccine hesitancy, resulting in lower vaccine hesitancy. Moreover, perceived susceptibility of the disease and perceived barriers of vaccination mediated the association between social media use and vaccine hesitancy.

**Conclusions:**

Our findings revealed a considerable level of COVID-19 vaccine hesitancy in South Korea. Gender-based and generation-based public health policies and communication are recommended. Efforts to lower the perceived risk of vaccine side effects and heighten perceived benefits of the vaccine are required. Although the use of media has a positive and negative effect on the population’s vaccine hesitancy, efforts should be made to disseminate reliable and timely information on media while confronting misinformation or disinformation for successive implementation of vaccine programs during pandemics.

## Introduction

Although the 2019 novel coronavirus (COVID-19) continues to spread worldwide and the public health emergency continues, the battle to overcome COVID-19 remains active. The development of effective vaccines has been highly anticipated, and several vaccines are now available; however, the timeliness of vaccine development and availability are not the only obstacles to overcome from a public health perspective. Raising vaccine acceptance and uptake among the public is essential to elevate public health emergency preparedness, which refers to the level of readiness for public health systems, communities, and individuals to prevent, respond to, and recover from public health emergencies [1]. A sizeable proportion of the population must be vaccinated to reach herd immunity and prevent the continued spread of the virus, and the goal of the South Korean government is to completely vaccinate 70% of the population [2,3]. According to a global survey collected in June 2020, which asked whether they would be vaccinated if the COVID-19 vaccine is proven safe, effective, and if available, 79.79% of the South Korean respondents responded positively [4]. However, there is evidence that the acceptance of the vaccine is declining [5]. A systematic review compared trends in vaccination receptivity over time, and a decrement of vaccine acceptance from >70% (March 2020) to <50% (October 2020) was observed [6].

Despite the availability of vaccination services, the phenomenon of delayed vaccine acceptance or refusal is referred to as vaccine hesitancy [7-9]. Numerous studies have attempted to define and categorize vaccine hesitancy and commonly propose that attitudes toward vaccination exist on a continuum of no demand to high demand and from accepting all vaccines to accepting no vaccines. Generally, vaccine-hesitant individuals are a heterogeneous group in the middle of this continuum [10]. Vaccine-hesitant individuals may refuse some vaccines but agree to others; they may delay or accept vaccines according to the recommended schedule but be unsure in doing so [11,12]. The Strategic Advisory Group of Experts Working Group on Vaccine Hesitancy proposed the determinants of vaccine hesitancy, which are categorized as follows: (1) contextual influences (eg, communication and media environment, historical influences, politics and policies), (2) individual and group differences (eg, personal, family and community members’ experience with vaccination, beliefs, attitudes about health and prevention, trust in health system, knowledge, perceived risk and benefits), and (3) vaccine-specific issues (eg, risk and benefit based on epidemiological and scientific evidence, introduction of a new vaccine, costs) [10].

Vaccine hesitancy is complex and context-specific, varying across time, place, and vaccines. Therefore, hesitation of COVID-19 vaccination should be understood in that context. Epidemiologically, COVID-19 is a highly infectious disease with a basic reproduction number of between 2 and 3, and there is a sharp increase in the number of confirmed cases worldwide [13]. COVID-19 vaccines were developed very shortly within a year, although it takes an average of 10.7 years to develop a new vaccine [14]. Further, a well-known aspect of the COVID-19 vaccination is that although the side effects occur less frequently, they can occur [15-17]. In this context, previous studies revealed the influence of individual and group differences on vaccine hesitancy such as perceived risk of COVID-19 infection [18,19], confidence in the capacity of health services to respond to the COVID-19 pandemic [20], and trust in authorities [21,22]. Vaccine-specific issues such as confidence in the efficacy of the vaccines [23-26], fear of side effects [19,25], and high conspiracy beliefs around the vaccines [27] were also revealed to predict COVID-19 vaccine hesitancy.

Along with the factors mentioned above, scholars paid attention to the effects of using various media (eg, offline media, online media, social media) as vaccine-related information sources on vaccine hesitancy. Vaccine-related health information has shaped perceptions, attitudes, and emotions related to the vaccine. Using offline media such as listening to the radio and reading the newspaper frequently was associated with increased vaccination odds [28,29]. Health information seekers who used newspaper articles as a vaccine-information source were more likely to perceive a vaccine as effective, being more likely to accept the influenza vaccine [30]. Refusing or delaying vaccinations of their children by parents was associated with using online media information sources [31,32]. Respondents among Medicare beneficiaries in the United States who relied on webpages for vaccine-related information were likely to be hesitant about COVID-19 vaccine uptake [33]. Social media platforms have become an increasingly popular source of health information, and growing interest has emerged in the role of social media in public health promotion. In particular, 2-way communication between health authorities and the public via social media is possible, and real-time exchange of health information among families and friends during a pandemic is possible [34-36]. The internet is widely used by authorities to inform the public about the latest news, disseminate public health knowledge, refute rumors, and facilitate effective coordination of medical, public, and pharmaceutical resources [37]. However, misinformation and rumors regarding COVID-19 vaccines have also emerged on social media platforms widely [38]. Engagement with vaccine-related information on social media was related to lower perceived vaccine efficacy [30], higher belief that vaccines are unsafe [39], higher conspiracy beliefs regarding the COVID-19 pandemic [40], and lower vaccination rates [34].

There is limited evidence about the acceptance or hesitancy of the COVID-19 vaccine in practice, and the influencing factors of vaccine hesitancy and whether and how media use can influence the public’s vaccine hesitancy warrants further exploration. Therefore, this study conducted a survey a month before the start of vaccination and addressed the level of vaccine hesitancy and investigated factors related to vaccine hesitancy along with which populations to prioritize in COVID-19 vaccination interventions. Moreover, we examined how media use interacts with psychological factors for vaccine hesitancy. Specifically, this study aimed to (1) examine the level of COVID-19 vaccine hesitancy, (2) quantify and test the relationships between sociodemographic, health-related, psychological factors, media use, and COVID-19 vaccine hesitancy, and (3) examine how psychological factors interplay with the media use on vaccine hesitancy. Implications for developing and implementing evidence-based interventions and policies to raise vaccine acceptance and uptake are also discussed in this paper.

## Methods

### Study Design and Sampling

We conducted a web-based cross-sectional survey on January 20, 2021—a month before vaccination was initiated in South Korea. The questionnaire, which consisted of 83 questions, was developed to (1) evaluate the public’s hesitancy of the COVID-19 vaccine and (2) assess the association with health-related factors, psychological factors, and media use by using an anonymous web-based questionnaire. The survey was conducted via a web-based platform from a research company called Korea Research. The company recruited participants by sending survey invitations containing general information about the survey, such as its aim and consent statement via email or text messages, and then registered survey panel members who met the inclusion criteria. The inclusion criteria were as follows: (1) 18 years or older, (2) a resident in South Korea, and (3) a Korean speaker. The company sampled the participants by age, sex, and geographic region–based proportional and quota sampling process. The respondents provided electronic informed consent that appeared on the first page of the survey, and the company protected the confidentiality of the anonymous respondents. Over 1033 participants completed the surveys, and 1016 were included in the analysis after excluding incomplete responses. This study was reviewed and approved by the Institutional Review Board at Seoul National University (IRB 2101/003-005), Seoul, South Korea. All participants provided their informed consent upon enrollment. The data collection took place over 5 days (January 20-25, 2021), a year after the Korea Centers for Disease Control and Prevention confirmed the first case at the early stage of the epidemic (January 20, 2020).

### Measurements

#### Dependent Variables

A 5-point scale questionnaire measured the intention to be vaccinated for COVID-19. Participants were asked, “If a vaccine for coronavirus (COVID-19) becomes available, would you want to receive it?” and provided response options “Definitely not,” “Probably would not,” “half and half,” “Probably would,” and “Definitely want to receive it.” The COVID-19 vaccination intention response options of “Definitely not,” “Probably would not,” and “half and half” were coded as “vaccine hesitancy=1,” and the options “probably would” and “Definitely want to receive it” were coded as “vaccine acceptance=0” to create dichotomous “hesitancy” versus “acceptance” variable.

#### Independent Variables

##### Sociodemographic Factors

Sociodemographic factors included gender (1=male, 2=female), age, family size (ie, living alone, more than 2 persons), the presence of children at home who attend school (more than one=1, none=0), marital status (ie, married, single, divorced, bereaved), and the participants’ residence (urban=1, rural=2). We also assessed education level (1=middle school or below, 2=high school graduate, 3=college and above) and monthly household income in South Korean won (1000 won=US $0.87; 1=<2 million won, 2=2 million to 3.99 million won, 3=4 million to 5.99 million won, and 4=6 million to 7.99 million won, 5=≥8 million won).

##### Health-Related Factors

Health-related factors included seasonal influenza vaccination history, presence of underlying disease, subjective health, and previous COVID-19 diagnosis for the participants. For seasonal influenza vaccination history, participants were asked, “Have you been vaccinated against the seasonal influenza flu in the last 5 years?” Responses included “every year,” “more than once,” “maybe once,” “never,” and “don’t know.” We grouped the participants as having seasonal influenza vaccination history (“every year,” “more than once,” and “maybe once”) or not (“never” and “don’t know”). Subjective health status (poor=1, moderate=2, good=3) was investigated to assess health-related factors. We also investigated the presence of underlying disease by asking participants to indicate all diagnosed underlying diseases (eg, hypertension, dyslipidemia, diabetes, chronic cardiac disease, asthma, cancer). We grouped the participants as being with or without diagnoses of one or more underlying diseases.

##### Media Used to Obtain COVID-19 Vaccination Information

We included the following question to assess participants’ media use to obtain vaccine-related health information via various information sources such as offline media (eg, television, radio, newspapers), online media (internet news sites, news portals), and social media (Twitter, Facebook, Kakao Talk, YouTube, blogs, communities). We used a 4-point rating scale (1=not at all to 4=always) to ask the following question: “How often do you use the following information source to seek information about COVID-19 vaccine?”

##### Psychological Factors Related to COVID-19 Vaccination

**Figure 1:**
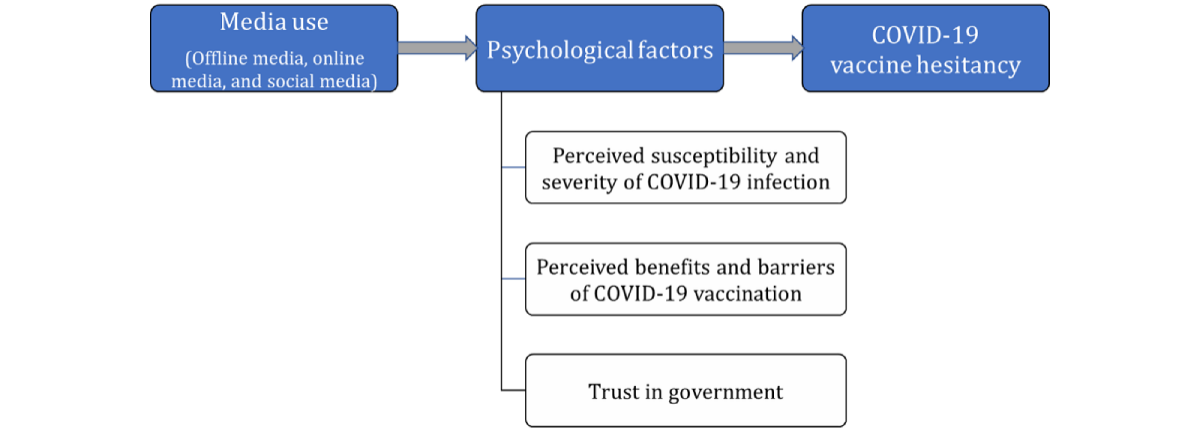
Framework of this study.

Questions to determine the psychological factors that could influence COVID-19 vaccination were adapted from the Health Belief Model and included perceived risk of COVID-19 infection, fear of COVID-19 infection, perceived benefits of COVID-19 vaccination, perceived barriers of vaccination, and trust in the government (Figure 1). Perceived risk of COVID-19 infection comprised 2 components: (1) perceived susceptibility, signifying an individual’s beliefs about their possibility of infection, and (2) perceived severity, signifying the seriousness of infection [25]. Participants were asked, “What do you think is the possibility of COVID-19 infection?” and “What do you think will be the severity if COVID-19 infects you?” Responses were rated on a 5-point rating scale, with “1=very low, 3=neither low nor high, and 5=very high.” Perceived benefits of COVID-19 vaccination were measured by 2 items measuring perceived chances of gaining specific benefits by COVID-19 vaccination: (1) self-protection of my health and (2) proved efficacy on preventing COVID-19 infection (1=extremely low to 5=extremely high; Cronbach alpha of .86). Two items addressed the perceived barriers of COVID-19 and included perceived chances of experiencing barriers against COVID-19 vaccination, such as (1) COVID-19 infection caused by vaccination and (2) concern about side effects of vaccination (1=extremely low to 5=extremely high; Cronbach alpha of .61). We also investigated participants’ trust in government by asking, “To what extent do you currently trust the government which respond to infectious diseases?” Responses were collected using a 5-point scale, with “1=extremely low to 5=extremely high.”

### Statistical Analysis

All quantitative variables were reported in numbers, proportions, means, and standard deviations. The responses to the COVID-19 vaccination acceptance questions were categorized into binary groups: (1) those who would accept COVID-19 vaccination (acceptance group) and (2) those who hesitated to uptake COVID-19 vaccination (hesitancy group). Differences in sociodemographic and health-related factors were compared with the COVID-19 vaccination hesitancy by using the chi-square statistics to determine the role of sociodemographic and health-related factors in COVID-19 vaccine hesitancy.

A multivariable analysis was developed in 2 stages. First, we performed a logistic regression to evaluate factors associated with COVID-19 vaccination hesitancy, including sociodemographic factors (ie, gender, age, family size, education, marital status, income, employment) and health-related factors (ie, subjective health and presence of underlying disease), media use (ie, offline media, online media, and social media), and psychological factors (ie, perceived susceptibility and severity toward COVID-19, perceived benefits and barriers of COVID-19 vaccination, and trust in government). In the second stage, a path analysis was carried out to describe the direct and indirect associations of media use and psychological factors with vaccine hesitancy using the Lavaan package (v0.6-9) [41] based on R version 4.0.5. The 3 types of media use (offline, online, and social media) were included in the path model at the same time to test the independent relations. Sociodemographic factors and health-related factors were added in the path model as control variables. Path models are a statistical method that, compared to multiple regressions, allow for the simultaneous assessment of several regression paths occurring between multiple dependent and independent variables and for the computing of direct, indirect (mediated), and total effects. Standardized parameter estimates were used to compare the magnitude of associations of the media use on mediators. Statistical analyses were conducted using R version 4.2 (R Foundation for Statistical Computing).

## Results

### Characteristics of the Survey Participants

Among the 1016 participants, 48.8% (496/1016) were men and 51.2% (520/1016) were women, with a mean age of 47.04 (SD 15.04) years (Table 1). The majority of participants had a family size of more than 2 persons (870/1016, 85.6%), and 59.9% (609/1016) were married. Half of the participants had at least some college education (532/1016, 52.4%), followed by those with only a high school education (456/1016, 44.9%). The most common monthly household income was approximately 2-3.99 million won (US $1688-US $3369; 360/1016, 35.4%), followed by 4-5.99 million won (US $3377-US $5057; 220/1016, 21.7%), and over 6 million won (US $5065; 52/1016, 2.4%) (Table 1). Among the participants, 87.3% (887/1016) lived in the urban areas, and about 22.2% (226/1016) had school-aged children. Additionally, 67% (681/1016) had received a seasonal influenza vaccination more than once in the previous 5 years, and 36.2% (368/1016) had more than one underlying disease. Approximately 35.5% (361/1016) reported their subjective health as good, 49.8% (506/1016) reported moderate, and 14.7% reported poor. Only 1.8% (18/1016) reported they had previously experienced COVID-19. Table 1 presents the characteristics of the sample population.

**Table 1:** Sociodemographic and health-related characteristics of the study participants (N=1016).

Characteristics				Values, n (%)
**Sociodemographic factors**				
	**Gender**			
		Male	496 (48.8)	
		Female	520 (51.2)	
	**Age (years), mean 47.04 (SD 15.04) years**			
		18-29	170 (16.7)	
		30-39	157 (15.5)	
		40-49	190 (18.7)	
		50-59	201 (19.8)	
		≥60	298 (29.3)	
	**Family size**			
		1 (living alone)	146 (14.4)	
		more than 2	870 (85.6)	
	**Marital status**			
		Married	609 (59.9)	
		Single/divorced/bereaved	407 (4.1)	
	**Presence of children**			
		None	790 (77.8)	
		More than 1	226 (22.2)	
	**Education level**			
		Middle school or below	28 (2.8)	
		High school graduate	456 (44.9)	
		College	468 (46.1)	
		Graduate school and above	64 (6.3)	
	**Income level (million won)^a^**			
		<2	229 (22.5)	
		2-3.99	360 (35.4)	
		4-5.99	220 (21.7)	
		6-7.99	155 (15.3)	
		≥8	52 (5.1)	
	**Residence**			
		Urban	887 (87.3)	
		Rural	129 (12.7)	
**Health-related factors**				
	**Seasonal influenza vaccination history (5 years)**			
		No	335 (32.9)	
		Yes	681 (67.0)	
	**Underlying disease**			
		None	648 (63.8)	
		More than 1	368 (36.2)	
	**Subjective health**			
		Poor	149 (14.7)	
		Moderate	506 (49.8)	
		Good	361 (35.5)	
	**COVID-19 experience**			
		None	998 (98.2)	
		Confirmed	18 (1.8)	

^a^Currency exchange conversion rate of 1000 won=US $0.87 is applicable.

### Vaccine Hesitancy

**Figure 2:**
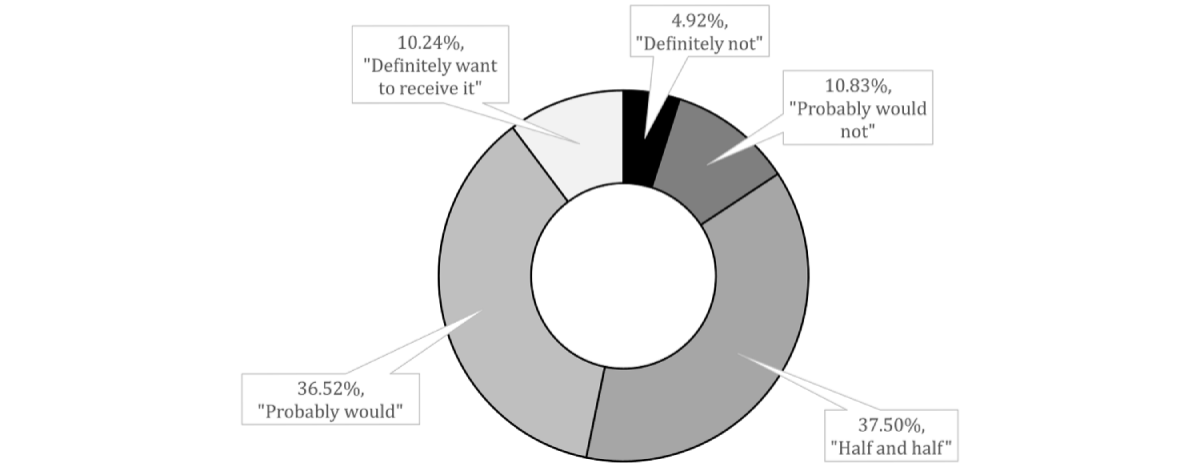
Distribution of responses regarding the question “If a vaccine for coronavirus (COVID-19) becomes available, would you want to receive it?”

Among the 1016 participants, 10.2% (104/1016) stated that they definitely would accept the vaccination, while 36.5% (371/1016) would probably accept the vaccination. However, 37.5% (381/1016) of the participants reported that they were “half and half,” 10.8% (110/1016) would “probably would not,” and 4.9% (50/1016) would “definitely not” get vaccinated (Figure 2). The COVID-19 vaccination intention response options of “definitely not,” “probably would not,” and “half and half” were grouped as “vaccine hesitancy” group (541/1016, 53.3%). The options “probably would” and “definitely want to receive it” were grouped as “vaccine acceptance” group (475/1016, 46.8%).

Differences in the sociodemographic and health-related factors were compared with the COVID-19 vaccination hesitancy by using chi-square statistics (Table 2). Women (*P*<.001) and unmarried participants (single, divorced, or bereaved) (*P*<.001) were more likely to demonstrate vaccine hesitancy. Group differences by age (*P*<.001) and monthly household income (*P*=.03) were statistically significant. Health-related factors such as having an influenza vaccination history (*P*<.001) and the presence of underlying disease (*P*=.003) were related to higher vaccine acceptance. However, group differences between participants’ different education levels and the presence of children were not statistically significant.

**Table 2:** Chi-square statistics for variables related to COVID-19 vaccination hesitancy and acceptance.

Characteristics				Likelihood of getting the COVID-19 vaccine (N=1016)					*P* value
				Acceptance (n=475), n (%)		Hesitancy (n=541), n (%)			
**Sociodemographic factors**									
	**Gender**							<.001	
		Male	272 (54.8)		224 (45.2)				
		Female	203 (39)		317 (61)				
	**Age (years)**							<.001	
		18-29	55 (32.4)		115 (67.6)				
		30-39	51 (32.5)		106 (67.5)				
		40-49	91 (47.9)		99 (52.1)				
		50-59	107 (53.2)		94 (46.8)				
		≥60	171 (57.4)		127 (42.6)				
	**Family size**							.89	
		1 (living alone)	69 (47.3)		77 (52.7)				
		More than 2	406 (46.7)		464 (53.3)				
	**Education level**							.97	
		Middle school or below	12 (42.9)		16 (57.1)				
		High school graduate	214 (46.9)		242 (53.1)				
		College	218 (46.6)		250 (53.4)				
		Graduate school and above	31 (48.4)		33 (51.6)				
	**Marital status**							<.001	
		Married	312 (51.2)		297 (48.8)				
		Single/divorced/bereaved	163 (40)		244 (60)				
	**Presence of children**							.39	
		None	375 (47.5)		415 (52.5)				
		More than 1	100 (44.2)		126 (55.8)				
	**Income level (million won)^a^**							.03	
		<2	103 (45)		126 (55)				
		2-3.99	164 (45.6)		196 (54.4)				
		4-5.99	113 (51.4)		107 (48.6)				
		6-7.99	80 (51.6)		75 (48.4)				
		≥8	15 (28.8)		37 (71.2)				
	**Residence**							.61	
		Urban	412 (46.4)		475 (53.6)				
		Rural	63 (48.8)		66 (51.2)				
**Health-related factors**									
	**Influenza vaccination history**							<.001	
		No	107 (31.9)		228 (68.1)				
		Yes	368 (54)		313 (46)				
	**Underlying disease**							.003	
		None	280 (43.2)		368 (56.8)				
		More than 1	195 (53)		173 (47)				
	**Subjective health**							.14	
		Poor	79 (53)		70 (47)				
		Moderate	223 (44.1)		283 (55.9)				
		Good	173 (47.9)		188 (52.1)				
	**COVID-19 infection experience**							.50	
		Not infected	468 (46.9)		530 (53.1)				
		Confirmed	7 (38.9)		11 (61.1)				

^a^Currency exchange conversion rate of 1000 won=US $0.87 is applicable.

### Media Used to Obtain COVID-19 Vaccination Information

We examined how often people used media sources (offline media, online media, social media) to learn about COVID-19 vaccination. Interestingly, participants sought little vaccine-related health information via social media; the average social media information seeking was close to “sometimes” (score=2) (mean 1.83 [SD 0.70]). By contrast, the participants used online media more often (mean 2.70 [SD 0.81]), followed by offline media (mean 2.66 [SD 0.95]) (Table 3).

**Table 3:** Media use and psychological characteristics of the study participants.

Characteristics					Values, mean (SD)
**Media** **use (4-point scale)**					
	Offline media			2.66 (0.95)	
	Online media			2.70 (0.81)	
	Social media			1.83 (0.70)	
**Psychological** **factors (5-point scale)**					
	Perceived susceptibility			3.05 (0.74)	
	Perceived severity			3.95 (0.76)	
	**Perceived** **benefits**			3.38 (0.81)	
		Self-protection of my health	3.74 (0.97)		
		Proved efficacy of the vaccine	2.80 (0.96)		
	**Perceived** **barriers**			3.29 (0.90)	
		Vaccination caused COVID-19 infection	2.85 (1.11)		
		Concerns about vaccination’s side effects	3.72 (1.01)		
	Trust in government			2.80 (0.68)	

### Psychological Factors Related to COVID-19 Vaccination

Participants perceived the risk of becoming infected with COVID-19 (perceived susceptibility) as being “moderate” (score=3) (mean 3.05 [SD 0.74]). Only 3.1% (31/1016) reported that perceived chance of infection is “very high” (score=5) and 18.8% (191/1016) reported “high” (score=4). Many participants reported that the chance of infection is “neither high nor low” (621/1016, 61.1%). The average perceived severity score was higher than perceived susceptibility, which was close to “high” (score=4) (mean 3.95 [SD 0.76]). However, among the participants, 55.1% (560/1016) reported that the severity would be “high” (score=4), and 22% (195/1016) reported “very high” (score=5). Participants’ perception of vaccination benefits was measured by 2 items measuring perceived chances of gaining specific benefits by COVID-19 vaccination. The average score was higher than “moderate” (score=3) (mean 3.38 [SD 0.81]). Among the benefits, “self-protection of my health” was the highest (mean 3.74 [SD 0.968]) and “proved efficacy of the vaccine” was the lowest (mean 2.80 [SD 0.964]). The average score for perceived barriers to vaccination was neither high nor low (mean 3.29 [SD 0.90]). The belief that a vaccination caused COVID-19 infection was relatively low (mean 2.85 [SD 1.110]); however, concerns about side effects were high (mean 3.72 [SD 1.007]). Among the participants, 23.8% (242/1016) reported their concerns that the vaccination’s side effects are “very high,” while 38.2% (388/1016) reported they were “high.” The average score of trust in government was slightly less than moderate (mean 2.80 [SD 0.68]) (Table 3).

### Factors Associated With COVID-19 Vaccine Hesitancy

Table 4 shows the results of the hierarchical logit regression models to test the association between vaccine hesitancy and participants’ sociodemographic factors, health-related factors, media use, and psychological factors related to COVID-19 vaccine hesitancy. A total of 60.5% of the variance was explained by the final model. The Nagelkerke R^2^ changes indicated that the incremental variances explained by each block of variables were 16.7%, 3.10%, and 40.7% for sociodemographic and health-related characteristics, media use, psychological responses, respectively. Out of the sociodemographic factors, female (odds ratio [OR] 1.967, 95% CI 1.36-2.86; *P*<.001), age in 40s (OR 0.467, 95% CI 0.23-0.95; *P*=.003), 50s (OR 0.47, 95% CI 0.23-0.96; *P*=.004), and over 60s (OR 0.49, 95% CI 0.24-0.99; *P*=.04) were significant individual predictors of COVID-19 vaccine hesitancy. Among health-related factors, participants who had seasonal influenza vaccination in 5 years (OR 0.50, 95% CI 0.34-0.75; *P*<.001) were less likely to hesitate to uptake the COVID-19 vaccine. Seeking COVID-19 vaccine-related information via social medias was related to higher tendency of vaccine hesitancy (OR 1.46, 95% CI 1.10-1.92; *P*=.01). Regarding psychological factors related to COVID-19 vaccination, among perceived risks of COVID-19 infection, higher perceived susceptibility was associated with lower vaccine hesitancy (OR 0.69, 95% CI 0.52-0.91; *P*=.01). Regarding the perceived benefits and barriers of COVID-19 vaccination, perceived benefits were related to lower chance of vaccine hesitancy (OR 0.007, 95% CI 0.05-0.10; *P*<.001) while perceived barriers (OR 1.63, 95% CI 1.29-2.07; *P*<.001) were related to vaccine hesitancy. Finally, lower trust in government was associated with vaccine hesitancy significantly (OR 0.72, 95% CI 0.53-0.98; *P*=.04).

**Table 4:** Factors associated with COVID-19 vaccination hesitancy^a^.

Characteristics		Odds ratio (95% CI)	*P* value
**Gender**			
	Male	Ref^b^	
	Female	1.967 (1.353-2.86)	<.001
**Age (years)**			
	18-29	Ref	
	30-39	1.052 (0.538-2.059)	.88
	40-49	0.467 (0.23-0.945)	.03
	50-59	0.47 (0.229-0.964)	.04
	≥60	0.49 (0.239-0.99)	.04
**Education level**			
	Under middle school	Ref	
	High school graduate	0.373 (0.12-1.162)	.09
	College	0.293 (0.092-0.929)	.04
	Graduate school	0.377 (0.099-1.438)	.15
**Income level (million won)^c^**			
	<2	Ref	
	2-3.99	1.114 (0.681-1.824)	.67
	4-5.99	0.871 (0.489-1.553)	.64
	6-7.99	0.923 (0.485-1.758)	.81
	≥8	2.09 (0.816-5.353)	.12
**Marital status**			
	Single/divorced/bereaved	Ref	
	Married	1.099 (0.642-1.882)	.73
**Presence of children**			
	None	Ref	
	More than 1	1.504 (0.853-2.652)	.16
**Residential area**			
	Urban	Ref	
	Town	1.324 (0.752-2.331)	.33
**Influenza vaccination history**			
	No	Ref	
	Yes	0.501 (0.337-0.746)	.001
**Underlying disease**			
	None	Ref	
	More than 1	1.213 (0.796-1.846)	.37
**Subjective health**			
	Bad	Ref	
	Moderate	0.893 (0.50-1.596)	.70
	Good	0.999 (0.533-1.872)	.99
**COVID-19 infection experience**			
	Not infected	Ref	
	Confirmed	3.419 (0.836-13.982)	.09
**Media use**			
	Social media	1.455 (1.101-1.922)	.008
	Online media	0.838 (0.616-1.139)	.26
	Offline media	0.939 (0.736-1.197)	.61
**Psychological factors**			
	Perceived susceptibility	0.685 (0.516-0.909)	.009
	Perceived severity	0.898 (0.679-1.187)	.45
	Perceived benefits	0.067 (0.045-0.099)	<.001
	Perceived barriers	1.631 (1.285-2.069)	<.001
	Trust in government	0.719 (0.528-0.978)	.04

^a^Nagelkerke R^2^=0.61.

^b^Ref: reference value.

^c^Currency exchange conversion rate of 1000 won=US $0.87 is applicable.

### Indirect Association Between Media Use and Vaccine Hesitancy Via Psychological Factors

**Figure 3:**
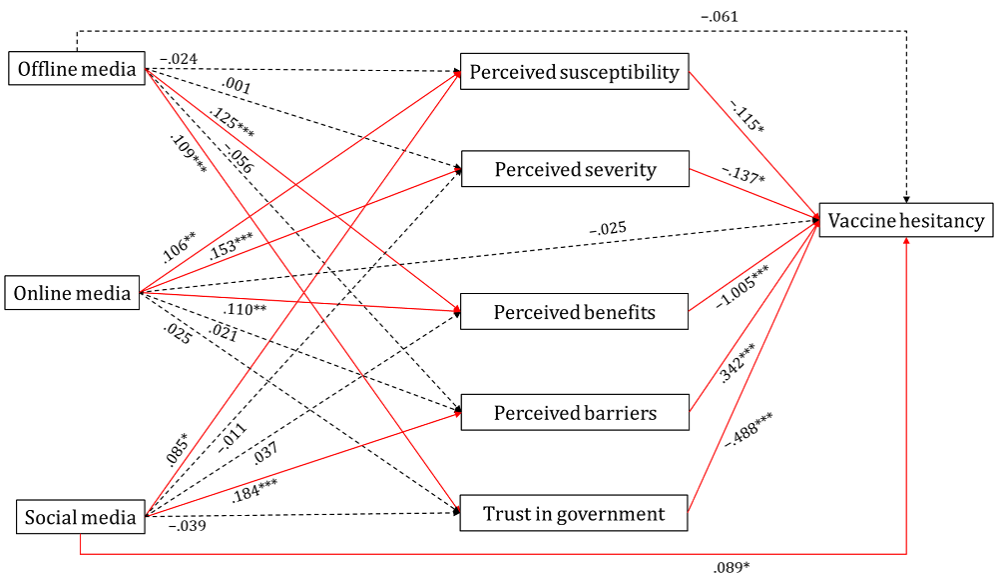
Path model showing the relationships between media use, psychological factors, and vaccine hesitancy. Note: Path coefficients are standardized regression weights. Nonsignificant paths are indicated as dotted lines. *P<.005, **P<.01, ***P<.001.

The results of the path model analysis showing the relationships between media use, psychological factors, and vaccine hesitancy are shown in Figure 3. Higher level of perceived susceptibility of COVID-19 was related with using online media (β=.106, *P*=.004) and social media (β=.085, *P*=.01) for vaccine-related information, while perceived severity of the disease was related to online media use (β=.153, *P*<.001). Regarding perceived benefits of COVID-19 vaccination, offline media use (β=.125, *P*<.001) and online media use (β=.110, *P*=.002) were related in a positive direction. Higher perception on barriers of vaccination was associated with social media use (β=.184*, P*<.001). Lastly, offline media use (β=.109, *P*<.001) was related to higher trust in government (Table 5).

We examined the standardized parameter estimates to compare the magnitude of associations of media use on vaccine hesitancy via psychological factors (ie, perceived susceptibility and severity of COVID-19 infection, perceived benefits and barriers of COVID-19 vaccination, and trust in government). Offline media use was related to lower vaccine hesitancy indirectly via higher perceived benefits and trust in government. With regard to online media use, the indirect association through perceived severity of COVID-19 infection and perceived benefits of vaccines were significant and related to lower vaccine hesitancy. Lastly, the indirect association between social media and vaccine hesitancy via perceived susceptibility (*P*=.01) of COVID-19 and perceived barriers of vaccines (*P*<.001) was significant. Interestingly, we found conflicting indirect relations between social media use and vaccine hesitancy. Although perceived susceptibility of COVID-19 infection negatively mediated the relationship of social media use with vaccine hesitancy, perceived barriers mediated the relationship of social media use positively.

**Table 5:** Standardized parameter estimates of indirect association between media use and vaccine hesitancy via mediators.

Independent variable, mediator		Vaccine hesitancy		
		Standardized parameter estimates	SE	*P* value
**Offline media**				
	Perceived susceptibility	0.003	0.004	.46
	Perceived severity	0.001	0.004	.99
	Perceived benefits	–0.126	0.031	<.001
	Perceived barriers	–0.019	0.013	.14
	Trust in government	–0.053	0.015	<.001
**Online media**				
	Perceived susceptibility	–0.012	0.007	.09
	Perceived severity	–0.021	0.01	.04
	Perceived benefits	–0.111	0.036	.002
	Perceived barriers	0.007	0.015	.62
	Trust in government	–0.012	0.017	.49
**Social media**				
	Perceived susceptibility	–0.01	0.006	.01
	Perceived severity	0.002	0.005	.77
	Perceived benefits	–0.037	0.037	.31
	Perceived barriers	0.063	0.016	<.001
	Trust in government	0.019	0.015	.22

## Discussion

### Principal Results

This study revealed a considerable level of COVID-19 vaccine hesitancy at 1 month before the start of vaccination in South Korea. Among the participants, 53.3% (541/1016) hesitated to uptake the COVID-19 vaccination, 37.5% (381/1016) reported they were “half and half” and 10.8% (110/1016) reported they would “probably not” or “definitely not” get vaccinated, while 46.7% (475/1016) of the participants would “likely” or “very likely” accept the vaccine. A decrement of vaccine acceptance was also observed in South Korea, as in other countries, from 79.8% (June 2020) to 46.7% (January 2021) [4-6]. This shows that efforts to increase vaccine acceptance are needed to meet the South Korean government’s target of 70% of the total population. Interventions and policies to raise vaccine acceptance and uptake are urgently needed.

Several additional findings are worthy of note. First, sociodemographic characteristics and health-related factors were standard subgroup variables cross-tabulated with vaccination hesitancy. Gender has emerged as a significant issue during this pandemic. Previous studies have shown that women are more likely to engage in preventive behaviors [42-44]; however, they are less willing than men to receive the vaccine [29], with more females declaring that they are unsure of taking the vaccine. Similarly, our study results reveal that women (224/1016, 45.2%) are more hesitant to receive COVID-19 vaccination than men (317/1016, 61%). Regarding age, higher levels of hesitancy among the participants in their 20s (115/170, 67.6%) and 30s (106/157, 67.5%) leave them particularly vulnerable to COVID-19. This result is similar to prior research investigating the relationship between sociodemographic factors and vaccine hesitancy during the COVID-19 pandemic [4,45,46]. Thus, gender-based and generation-based public health policies and communication are recommended.

Second, the results revealed the association between psychological factors and vaccine hesitancy. Perceived barriers such as concerns about side effects caused by a COVID-19 vaccine had a significant and robust association with vaccine hesitancy. The perceived susceptibility of COVID-19 infection and benefits of vaccination (eg, proven efficacy of COVID-19 vaccines) related negatively to vaccine hesitancy. Trust in government was also negatively associated with vaccine hesitancy. The predictive power of psychological factors on vaccine hesitancy has been emphasized by numerous studies [21,47,48]; similarly, psychological factors explained 40.7% of the variance for vaccine hesitancy in this study. Therefore, interventions and communication strategies aimed to improve perceived benefits of vaccination and reducing perceived barriers to vaccination will be useful in responding to vaccine hesitancy [48]. A well-known aspect of COVID-19 vaccination is that it may induce adverse events. According to a report of COVID-19 vaccine safety monitoring in South Korea, 16,196 adverse events (0.5%) following 3,586,814 administered doses of COVID-19 vaccines were reported in approximately 2 months (February 26 to April 30, 2021) [49]. Of these, 15,658 (96.7%) were nonserious adverse events comprising local and systemic reactions, including myalgia, headache, fever, and pain at the injection site; however, 538 (3.3%) were serious adverse events, including 73 (0.5%) deaths. These results are similar to the findings of the clinical trial [50]. Although possible side effects are comparatively rare, the risk of their occurrence may significantly influence the choice to not vaccinate. The decision to vaccinate (or not) shares many common points with one’s chosen level of self-protection [51]. The most obvious link between the two problems is that the decision maker’s objective is to reduce the probability of an undesirable event. Vaccination reduces the probability of the primary disease; however, another cost may induce other new risks. From this point of view, the decision-making on vaccination is a trade-off between the risk of the primary disease and the risk of incurring side effects [52]. Therefore, the vaccine hesitancy due to the perceived barrier such as concerns on vaccine-induced side effects can be regarded as a risk aversion [53,54]. Moreover, in Korea, face masks were promoted based on substantial relative benefits, high efficacy of slowing viral spread, and low cost. Several studies report high compliance and high efficacy beliefs on wearing masks among the Korean population [42-44]. Therefore, people might prefer to wear face masks over vaccination to avoid the risk of the side effects from vaccination.

Third, this study suggests that the use of media to obtain vaccine-related information has a positive or negative relation with the population’s vaccination decision-making. The mechanisms by which psychological factors mediate the association between media use and vaccine hesitancy were also revealed. Offline media such as TV, radio, and newspapers were associated with higher perceived benefits of a COVID-19 vaccine and higher trust in government, which led to lower vaccine hesitancy. Kim and Jung [28] suggested that appropriate use of offline media (eg, radio and newspapers) is critical to increase the population’s vaccination rate and is a way to reduce the information gap among social classes. Online media also had an indirect association with vaccine hesitancy in a negative direction, mediated through perceived severity of COVID-19 infection and perceived benefits of the vaccine. This result is contradictory with that reported in other studies reporting the association between online media use and higher vaccine hesitancy [31-33]. Therefore, further studies to examine the role of online media on vaccine hesitancy are needed.

Regarding social media, frequent social media use was related to vaccine hesitancy even when other types of media use (eg, offline, online media) and sociodemographic, health-related, and psychological factors were included in the model. Social media use was related with vaccine hesitancy via perceived susceptibility in a negative direction and perceived barriers in a positive direction. This result implies that social media as a health information source could act as a double-edged sword. Social media has been criticized because it propagates more misinformation than any other media type [55] and induces a high level of anxiety, depression, and even conspiracy beliefs during the COVID-19 pandemic [56,57]. The vaccine hesitancy owing to false beliefs caused by controversies and misinformation in social media is well documented in the literature [30,39,58,59]. Nevertheless, social media can also be used to build a positive perception of vaccination [30] and disseminate valuable evidence-based health information and recommendations rapidly to many people timely [60]. The quality and reliability of the information provided via social media should be improved [34,61]; public health communicators are encouraged to establish a web-based reputation as experts worth following or visiting online [30].

### Implications

A set of implications for interventions, communication strategies, and future research can be drawn from this study. Since this study was conducted a month before the start of vaccination, it provides a valuable opportunity to understand the public’s responses on novel vaccines and develop practical implications for implementing effective vaccination programs in a future epidemic. First, as the perceived barrier was the highest predictor of vaccine hesitancy, efforts to lower the perceived risk of vaccine side effects and heighten perceived benefits of the vaccine are required. This study indicates that social media is related to heightened perceived barriers, thereby leading to vaccine hesitancy, which is not very surprising. The phenomenon of “infodemics,” defined as the rapid spread and amplification of vast amounts of valid and invalid information on the internet or through other media, is a tremendous and ongoing challenge in the COVID-19 pandemic [62]. Communication efforts of public health authorities to provide accurate and reliable information, confront misinformation or disinformation, and reduce the negative impact of such infodemics are required. Eysenbach [63] recommended in his commentary to promote (1) information monitoring, (2) health and science literacy skills, (3) fact checking or peer review of the information, and (4) timely and accurate knowledge transition to fight the infodemics. Using alternative communication tools can provide official messages from the public health authorities to the public. For instance, emergency alert text messages [44] or official social media accounts [37] could be considered. Second, building trust in the government and public health authorities offers another solution. Trust is an essential factor influencing people’s perception and behavior during a pandemic and influences people’s willingness to be vaccinated [64,65]. Trust in institutions or persons decreases the perceived risk of new technologies and indirectly impacts the higher risk acceptance of the new technologies [64,66]. As the COVID-19 vaccine is newly developed and the development period was short, the public might recognize the COVID-19 vaccine as new technology. Therefore, it could be assumed that the public trust in governments can encourage the public to accept the risk of adverse events induced by COVID-19 vaccination. Trust is critical, and it is more easily broken and difficult to maintain under high-risk and high-uncertainty situations [67].

### Limitations

This study has several limitations worthy of note. First, although there are several types of social media such as official social media, professional social media, and public social media [37], this study did not examine the effect of each type of social media. Further studies should examine the effect of each type of social media and whether the effect is mediated via psychological factors. Second, we were not able to investigate the potential psychological factors proposed by previous studies such as subjective norms [68,69], knowledge [70], or contextual barriers such as accessibility [71]. Finally, since this study was designed cross-sectionally, we identified the associations between media use, psychological factors, and vaccine hesitancy rather than causal inference.

### Conclusions

This study’s findings suggest that social media plays a role in disseminating vaccine-related information, especially vaccination benefits, which can help the public accept the COVID-19 vaccine for disease control. Conversely, social media can also increase concerns about a vaccination’s side effects, stimulating vaccine hesitancy. Sociodemographic factors such as gender and age should be considered in public health interventions, and greater attention should be given to the younger adults and females during a pandemic for effective vaccination campaigns. This study highlights the need for government and public health authorities to consider approaching their public with additional efforts to promote acceptance of the COVID-19 vaccine. Efforts to disseminate reliable and timely information and monitor social media misinformation are needed during the pandemic. Understanding the intentions of vaccination and factors associated with the intentions may help inform public health authorities about evidence-based interventions; vaccination strategies are necessary to achieve broader community uptake.

## Abbreviations

OR: odds ratio
